# Cancer, immunity and inflammation. Report from the CDD Cambridge Conferences 2018 and 2019

**DOI:** 10.1038/s41419-019-2032-0

**Published:** 2019-10-22

**Authors:** Gianmaria Liccardi, Francesca Pentimalli

**Affiliations:** 1 The Breast Cancer Now Toby Robins Research Centre, Institute of Cancer Research, Mary-Jean Mitchell Green Building, Chester Beatty Laboratories, 237 Fulham Road, London, SW3 6JB UK; 2 Cell Biology and Biotherapy Unit, Istituto Nazionale Tumori IRCCS, Fondazione G. Pascale, Naples, 80131 Italy

**Keywords:** Cancer microenvironment, Cancer therapy, Inflammation

The Medical Research Council, Toxicology Unit and CDD*press* (representing the CDDifferentiation, CDDisease, CDDiscovery journals, Springer Nature) organized the *‘Genes versus environment in cancer*’ and the *‘Cancer, Immunity & Inflammation*’ conferences, which were held in September 2018 and 2019, respectively, in Cambridge at the stunning Clare College, founded in 1326 along the river Cam. The conferences focused on the complex interplay of factors that contribute to cancer susceptibility, development, and resistance, providing an excellent discussion forum on the key challenges and the latest advancements that mostly promise to propel this field forward, hosting high-profile speakers.

**Ashok Venkitaram** (Cambridge, UK) showed that toxic agents such as formaldehyde and acetaldehyde, pervasively found in the environment but also endogenously accumulated in certain tissues as byproducts of cellular metabolism, are able to lower the abundance of BRCA2. As a consequence, these agents induce haploinsufficiency, particularly in individuals carrying BRCA2 heterozygous mutations, in which the threshold of a functional protein is lower than in wt carriers, thereby promoting spontaneous mutations and tumorigenesis. This explains why BRCA2 loss affects specific tissues, setting the bases for possible chemopreventive strategies in mutation carriers, and impacts on public health and safety issues alerting on the detrimental effects of widespread environmental and dietary aldehydes.

**Vishva Dixit** (San Francisco, USA), who was awarded the **CDD Juerg Tschopp Prize 2018**, discussed a more recently identified cancer predisposing gene, the *BAP1* tumor suppressor. *BAP1* germline mutation predisposes carriers to the development of mesothelioma, uveal, and cutaneous melanoma and various other tumors, a condition known as BAP1 cancer syndrome. *BAP1* depletion, however, triggers cell death in many cell types, which could seem paradoxical considering its tumor suppressor function. In his engaging Keynote Lecture Dixit clued in on this seeming paradox showing that BAP1, which encodes a deubiquitinase, functions in concert with ring finger protein 2 (RNF2) to fine tune the activity of histone H2A. While RNF2, through H2A monoubiquitination, silences the *BCL2* and *MCL1* prosurvival genes and thereby induces apoptosis in some cell types, this does not occur in melanocytes, suggesting that BAP1 promotes tumorigenesis in cells that do not engage such RNF2 apoptotic programme.

Moving to the p53 tumor suppressor, **Karen Vousden** (London, UK), who was awarded the **CDD Award 2018**, retraced the long road leading to her key discoveries of p53 functions. She highlighted how the identification of the p53 target gene *TIGAR* led to define p53 role in regulating cell metabolism, showing that p53 can also protect cells rather than killing them upon stress conditions. Vousden showed how the ability of p53 to rewire cell metabolism, limiting ROS and providing nutrient sources, can be detrimental favoring cancer cell survival. Indeed, some tumor derived p53 mutants selectively retain these functions. She laid the ground for the development of novel tailored nutritional approaches, which might aid conventional cancer treatments.

Similarly, **Ivano Amelio** (Cambridge, UK) showed that p53 mutants can interact with the hypoxia inducible transcription factor HIF1 promoting the expression of a gene set that generates a pro-tumorigenic extracellular microenvironment in lung cancer.

**Xin Lu** (Oxford, UK) showed how p53 transcriptional activity can be modulated by the direct binding of iASPP, which affects specific p53 targets. Interestingly iASPP, mostly known as an oncogene able to inhibit the p53 apoptotic function, was also found to act as a tumor suppressor in certain microenvironmental contexts.

Finally, **Carol Prives** (New York, USA) received the **2019 CDD Award** presenting her work on the p53 control of the mevalonate pathway via the sterol regulatory element-binding protein 2, which represents an early event for hepatocellular carcinoma (HCC) development. She also discussed the role of MDM2 in promoting ferroptosis independently of p53, likely through PPARα.

Still on HCC, **Michael Karin** (La Jolla, USA) presented a novel substrate of caspase 2 promoting steatosis and nonalcoholic steatohepatitis progression, identifying in caspase 2 a new target to prevent and treat these diseases.

**Tak Mak** (Toronto, Canada) provided an insightful and compelling argument about the current state of cancer therapy, summarizing collective translational efforts and the lesson learned so far from common experience. He showed how crucial is the activation of T cells to mount an immune response against cancer cells and how this can be boosted with immunotherapy approaches based on immunecheckpoint inhibitors (using CTLA-4, PD1, and PD-L1 inhibitors); he finally discussed his most recent work on choline acetyltransferase-expressing T cells that are required to control chronic viral infection.

A new approach based on the stimulation of the immune system was proposed by **Scott W. Lowe** (New York, USA): a rational combination of drugs can be used to counteract lung cancer by inducing cell senescence and, in particular, a senescence associated secretory phenotype that is able to invoke the attack of NK cells within the microenvironment stimulating the antitumoral immune surveillance.

**Doug Green** (Memphis, USA) showed how targeting autophagy can be used as another strategy to enhance anticancer immunity. LC3-associated phagocytosis in the myeloid compartment can tackle tumor growth via the upregulation of the cGAS-STING pathway.

**Yufang Shi** (Shangai, China) discussed the role of mesenchymal stromal/stem cells (MSCs) in cancer. MSCs have the ability to modulate the microenvironment and control tumor growth and metastasis orchestrating both innate and adaptive, local, and systemic immune responses.

**Ying Wang** (Shangai, China) presented recently identified data on how macrophages, key players of both the innate and adaptive immune responses, can be trained to become anti-inflammatory. She showed in particular that IGF2 can program macrophages during their maturation determining a metabolic commitment for OXPHOS, which shapes their anti-inflammatory competences. Adoptive transfer of such reprogrammed macrophages functioned in a mouse model of autoimmune disease and promises to be relevant in other inflammatory conditions.

**Charles Swanton** (London, UK) presented his studies on cancer evolution discussing the importance of mutations within tumours and how the targeting of trunk mutations that are communal to the main bulk of the tumour cells originate the subsequent heterogeneity in the resistant cancer cells. Intratumoral heterogeneity and genomic instability are finely tuned allowing cancer cells to evolve during the disease course evading immune surveillance and resisting to therapies. Targeting such heterogeneity and new clonal antigenic architecture might help to reduce the treatment failure associated with targeted monotherapies.

Another key mechanism allowing tumor cells to adapt to environmental stress factors such as hypoxia, starvation, oxidative, or genotoxic stress was discussed by **Paul Sorenson** (Vancouver, Canada): cancer cells, upon stress cues, initiate a translational reprogramming at the mRNA level that inhibits overall translation activity to preserve energy and nutrients and induces at the same time the expression of major stress adaptor proteins (eIF2alpha, mTORC1, and EF2K) that allow them not only to survive but also to evolve to more aggressive phenotypes.

Conversely, **Pierre Close** (Liege, Belgium) proposed a new strategy to counteract melanoma cell survival and resistance to therapy by acting on the protein synthesis rewiring mediated by wobble tRNA modification enzymes, which are required by cancer cells for specific decoding during mRNA translation.

For his pioneering studies defining key components of cell death and necroptotic pathways, **Xiadong Wang** (Bejing, China) was awarded the **CDD Juerg Tschopp Prize 2019**. He introduced the audience to a new mechanism leading to apoptosis execution. He showed that estrogen and its related steroid hormones at the high concentration reached in the developing placenta, induce apoptosis by binding to phosphodiesterase 3A (PDE3A), which recruits and stabilizes the Schaflen 12 (SLFN12) protein increasing its levels. SLFN12 then binds to ribosomes impairing the engagement of signal recognition particles thereby blocking the translation of antiapoptotic BCL2 and MCL1, the decrease of which triggers apoptosis. This process guarantees the dynamic cell turnover occurring in the development of placenta that is required for a successful implantation.

Cell death pathways were discussed further with other engaging talks including: **Vishva Dixit** presenting data from a catalytic dead caspase 8 knock-in mouse, which revealed an unexpected cross talk between apoptosis, necroptosis, and other death pathways; **Andrew Oberst** (Seattle, USA) showing a recent strategy devised to induce necroptosis in cancer cells acting on the RIP kinases to achieve tumor clearance through the induction of antitumor immunity unleashed by dying cells; **Peter Vandenabeele** (Gent, Belgium) reporting a thorough comparative analysis of immunogenic cell death triggered by necroptosis, apoptosis, and ferroptosis; **Pascal Meier** (London, UK) showing how the ubiquitin signaling system impacts on TNF-induced cell death regulating RIPK1 activity and how such modulation could be used to limit inflammation in clinical applications based on TNF administration.

Finally, the 2018 Nobel Laureate **Greg Winter** (Cambridge, UK) provided a delightful end to the 2019 meeting describing his successful approach to generate a diversity of antibodies through the phage display method inspired by the principle of genetic change and selection to evolve new proteins. Spanning from the first humanized antibodies to the latest bicycles peptides, he described all the steps that have led so far to the development of a wide range of successful therapeutics, highlighting pros and cons of each class as well as challenges for future developments.

Despite the intense schedule of inspiring talks, the meetings provided many opportunities for discussions and interactions with leading scientists from around the globe in a very pleasant atmosphere. During the conferences the Editorial Boards of the three Cell Death journals gathered to discuss editorial strategies along with Springer Nature representatives who showed the performance of the various journals. Gerry Melino, founder and chief editor of CDD, now in its 25th anniversary, encouraged all editors to keep working to the highest publishing standards providing a high quality and timely service to the whole scientific community to which the journals belong.

